# Kinesiophobia as a Barrier to Symptom Management Using Physical Activity When undergoing Cancer Therapy: A Preparatory Study Describing Patients’ Experiences With the New Instrument Tampa-Scale for Kinesiophobia-Symptoms and Interviews

**DOI:** 10.1177/15347354241303454

**Published:** 2024-11-29

**Authors:** Tove Bylund-Grenklo, Anna Efverman

**Affiliations:** 1 University of Gävle, Gävle, Sweden

**Keywords:** cancer nursing, cancer rehabilitation, emesis, exercise, fear of motion, integrative cancer therapies, oncology nursing, physical activity, symptoms

## Abstract

**Background:** Cancer care professionals need to be aware of kinesiophobia, fear of motion, in patients undergoing cancer therapy. The new instrument the Tampa-Scale Kinesiophobia Symptoms (TSK-Symptoms) aims to measure fear and avoidance of motion in relation to multiple symptoms (eg, nausea, fatigue, anxiety, pain). It is modified from the TSK, which relates solely to pain. **Aim:** To test the feasibility of the TSK-Symptoms, to quantify kinesiophobia in patients with cancer, to study whether kinesiophobia was associated with symptoms or physical activity, and through interviews to gain a deeper understanding of patient experiences. **Methods:** In this preparatory longitudinal study, patients (n = 55, mean age 68 years; 51% men; 38% had prostate cancer, 23% breast cancer) undergoing radiotherapy provided questionnaire data on kinesiophobia using the new instrument TSK-Symptoms, symptoms and physical activity twice (at baseline, T1, and 1 week later, T2). Eight patients were interviewed. **Results:** At T1 and T2, 4 of 54 (7%) and 8 of 55 patients (14%) reported kinesiophobia (*P* = .009). From T1 to T2, occurrence of nausea increased. Of the 16 nauseated patients at T2, 6 (38%) reported kinesiophobia compared to 2 (5%) of the 39 nausea-free patients (*P* = .005). Patients who reported kinesiophobia practiced less physical activity (median 0 days at moderate intensity at T1 (*P* < .001), median 2 days at moderate intensity at T2, *P* = .006) compared to patients free from kinesiophobia (median 4 and 5 days). Three qualitative content analysis categories described patient experiences: (1) “Struggling to stay physically active in an extraordinary situation associated with burdensome symptoms,” (2) “Feeling damaged and at the same time grateful,” and (3) “Needing support due to fear of motion and of worsened condition.” **Conclusions:** This preparatory study showed that the new instrument the TSK-Symptoms was feasible for use in patients undergoing cancer therapy to quantify kinesiophobia, which was present in approximately 1 in 10 patients. Kinesiophobia was more common in patients with nausea, and patients reporting kinesiophobia practiced less physical activity. Patients highlighted a need for support. The psychometric properties of the TSK-Symptoms, completed on several languages, need to be evaluated. Cancer care professionals may quantify kinesiophobia using the TSK-Symptoms instrument and give kinesiophobic patients support.

## Introduction

It is not known whether cancer kinesiophobia, which means excessive fear of motion,^1^ occurs in relation to multiple symptoms^2^ during cancer therapy since this seems to be a novel research area.^3,4^ Various symptoms, for example, pain,^5-9^ fatigue,^10-14^ and lymphedema^15-18^ have been reported to be associated with developing kinesiophobia. Since patients usually experience a multitude of simultaneously occurring symptoms during cancer therapy,^2^ an instrument for quantifying kinesiophobia in relation to multiple symptoms seems to be needed.

Kinesiophobia may be defined as “an excessive, irrational and limiting fear of motion and activity”^1^ resulting from a feeling of vulnerability to injury or re-injury. Originally, the feeling of vulnerability was thought to be related to painful injury.^1,19,20^ The fear-avoidance model,^19,20^ frequently adopted to explain kinesiophobia in non-cancer populations,^19,20^ suggests that it is not only the severity of symptoms, but also the patient’s thinking patterns that affect the experience of symptoms. Negative thoughts about symptoms, that is, “catastrophizing thoughts,” create fear and reduce physical activity.^20^ Originally, the model proposed that fear avoidance occurred specifically in relation to pain.^1,19,20^ However, cancer care professionals may hypothesize, based on clinical experience, that fear avoidance may occur in relation to multiple cancer-related symptoms.^2,4^ For example, a nauseous patient undergoing emetogenic cancer therapy^21,22^ may have negative thoughts, that is, “catastrophizing thoughts,”^20^ that vomiting will occur when being physically active. According to the fear-avoidance model, catastrophizing thoughts and fear of motion may induce avoidance of motion, consequently not using the body for what it is made for (disuse), which leads to functional impairment (disability).^20^ For example, catastrophic thinking regarding falls is rather common in older frail populations, predicting physical inactivity.^23^ The absence of catastrophic thoughts tends instead to cause the patient to confront and expose him-/herself to the threat, that is, motion and physical activity, which may relieve symptoms and prevent functional limitations.^20,21^

Physical activity is an integrative therapy having the potential to reduce cancer-related symptoms^24^ and even increase survival,^25^ but adherence to practicing physical activity tends to be poor.^26^ A recent meta-analysis of 64 studies highlighted an association between psychosocial factors, such as behavioral control and attitudes, and physical activity adherence. Studies regarding the significance of kinesiophobia for physical activity adherence in patients with cancer were pointed out to be lacking.^26^ Notably, cancer care professionals either seem to be unaware of kinesiophobia as a potential barrier to physical activity during cancer therapy^27-30^ or suggest that kinesiophobia is a barrier, but one that seems difficult to quantify.^4^ Health professionals had doubts concerning how to treat cancer survivors, for example how to support physical activity.^28,29^ In non-cancer populations kinesiophobia acted as a barrier to physical activity.^1,19,20,23^ Further, decliners of a physical activity program during chemotherapy for cancer reported more kinesiophobia in relation to fatigue than patients who adhered to the program,^13^ indicating the value of further studying kinesiophobia in relation to multiple symptoms experienced when undergoing cancer therapy.

The difficulties and possibilities of quantifying kinesiophobia have been highlighted by others.^3,4^ Studies have observed kinesiophobia in relation to single symptoms previously, for example pain following breast cancer surgery,^6,7,9^ insertion of implantable venous access ports,^8^ temporomandibular pain,^31^ fatigue^10-13^, or lymphedema.^15-18^ However, patients during cancer therapy commonly suffer from a multitude of simultaneous occurring symptoms,^2,21,22^ which limits the implications of these studies for the highly heterogenous target population of patients undergoing various cancer therapies. The Tampa-Scale for Kinesiophobia (TSK)^32,33^ and the TSK version Chronic Fatigue Syndrome^8^ unfortunately do not measure kinesiophobia in relation to multiple symptoms,^2^ making these methods less applicable in clinical standard care. The field of integrative cancer care lacks knowledge about whether kinesiophobia occurs in patients during cancer therapy in relation to multiple symptoms.

## Aim

Prior to a full-scale multi-center study of symptoms, physical activity, kinesiophobia and quality of life, the present preparatory study aimed to test the feasibility of our new instrument TSK-Symptoms applied to quantify kinesiophobia in relation to multiple symptoms in patients with cancer, to study if kinesiophobia was associated with symptoms or physical activity practice, and through interviews to provide a deeper understanding of experiences on kinesiophobia, cancer-related symptoms and physical activity.

## Methods

### Study Design and Context

This preparatory study^34^ used a descriptive longitudinal design, collecting quantitative questionnaire data and qualitative interview data within an ordinary cancer care setting. The study was conducted at the radiotherapy unit of one Swedish oncology clinic preceding a full-scale multi-site study (sample size calculation was only made for the full-scale study, not for the preparatory study presented here). The head of the department and the regional ethical committee (ethical approval number Linköping 2015/101-31, dates 2015-03-25 and 2016-04-12) approved the questionnaire data collection, and the interview data collection was conducted within development work of standard care.

### Inclusion

**Figure 1.**
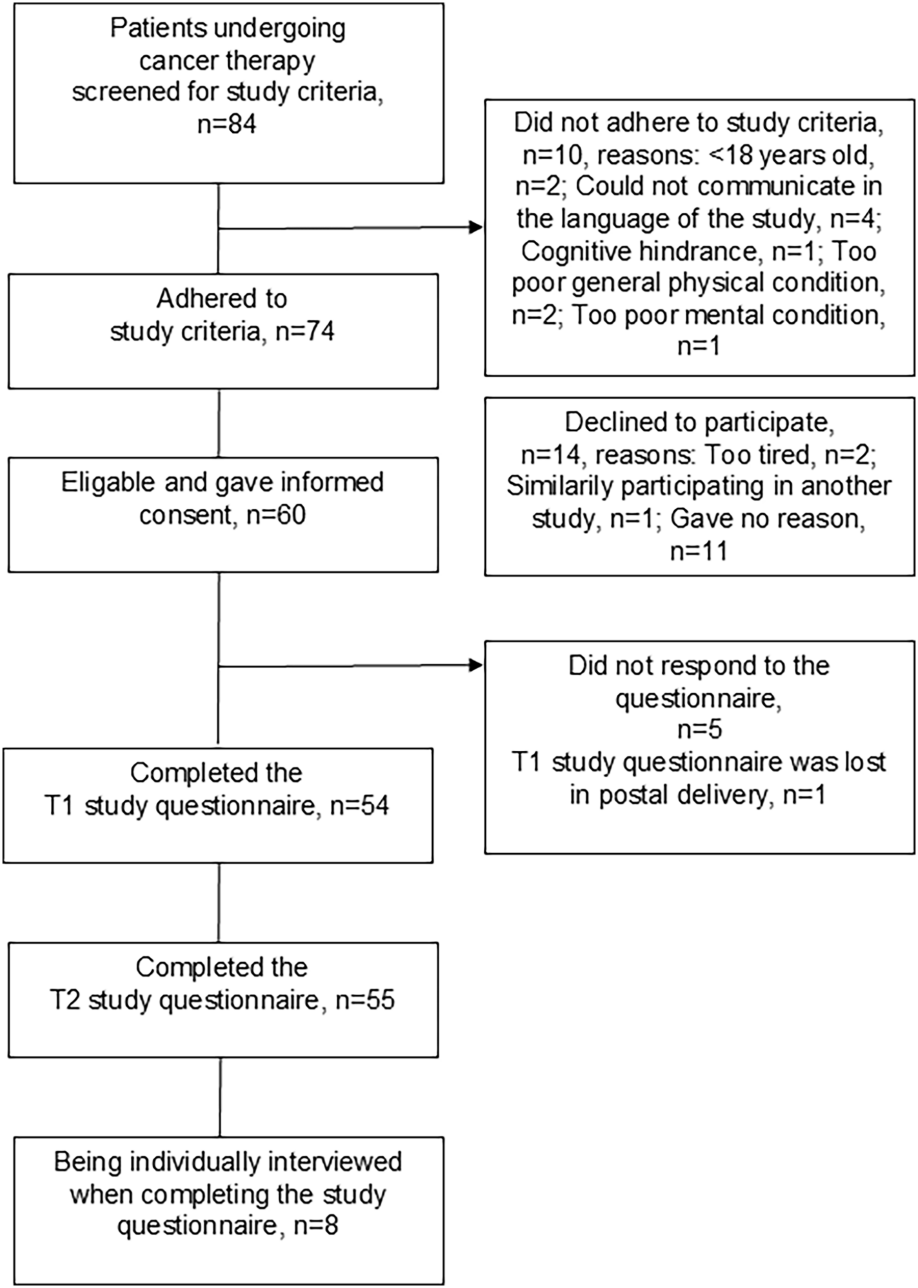
Flow-chart of patients included and contributing to the data collection procedure adopting quantitative questionnaires and qualitative interviews.

The day preceding a randomly selected study day, standard care radiotherapy nurses consecutively, according to a standardized protocol covering the study criteria, screened all patients who were scheduled to receive external radiotherapy for cancer during the study day, and provided written detailed information and short oral study information. They asked, using their own words: “May I provide you with information regarding a study on activities, symptoms, and quality of life?” They stated that participation was voluntary and that they would ask the day after if the patient wanted to participate, and that all responses will be sent to the study evaluator and not to be read by the standard cancer professionals. Inclusion criteria were patients undergoing external fractionated radiotherapy for cancer irrespective of type or stage of cancer or concomitant cancer therapies, who were at least 18 years of age and had the physical, mental and linguistic capacity to give their informed consent. Exclusion criterion was patients who received their very first radiotherapy session on the day for T1 (ie, this criterion excluded all patients given a single radiotherapy fraction with palliative intention). Of 84 patients scheduled for radiotherapy at the study day, 72 adhered to the study criteria, and 57 gave informed consent prior to participation (Figure 1).

### Data Collection

Data was collected longitudinally using a study questionnaire twice, at Time 1 (T1), after receiving an accumulated dose of mean 28.9 Gy ± Standard Deviation (SD) 18.5 Gy of the total dose 57.7 + 16.3, and at Time 2 (T2) 1 week later (after receipt of additional 5 fractions). The radiotherapy nurses delivered simultaneously the T1 and the T2 questionnaires to the included patients by using separate envelopes, to be opened on the day for T1 (at the study day) and the day for T2 (1 week later). The participants completed the questionnaires in private (mostly at home or at the patient hotel/ward unit) either in writing, using “pen and paper,” or using a digital web-based questionnaire service (they were free to choose method of response). Code numbers were used to allow the evaluator to connect the T1 and T2 answers from one and the same patient, without using any personal information on the patients. The participants returned the written questionnaires to the independent evaluator in designated response boxes at the radiotherapy department, or by post using pre-paid envelopes. The standard care radiotherapy nurses did not handle any responses, to secure confidentiality and avoid therapist-based bias. In the absence of a response within approximately 10 days after T1 and T2, respectively, study coordinating nurses made one telephone call to remind the patient. The study questionnaire covered the below described areas. At the end of the questionnaire, the patients were able to make free text comments, using their own words, regarding the relevance and wording of the questionnaires.

Clinical, sociodemographic, daily activity and quality of life background variables: The radiotherapy nurses collected data on the cancer diagnosis, irradiated field and dose of radiotherapy from the patients’ medical records. The T1 questionnaire covered questions regarding sociodemographics (eg, age, sex, marital status, educational level, and occupational status; the variables are seen in Table 1). The T1 questionnaire also covered daily living activity: “How many of your usual daily activities have you been able to perform the last week?” (7-graded category scale from “No activities” to “All activities”),^21^ and overall Quality of Life (QoL); “Overall, how did you experience your quality of life?” (8-graded scale from “0; Worst imaginable” to “7; Best imaginable”).^35^

**Table 1.** Demographics and Clinical Characteristics, Activity, and Perceived Quality of Life.

Variable	Patients (n = 55)
Gender, n (%)	n = 55
Man	28 (51)
Woman	27 (49)
Age in years, mean ± SD	67.5 ± 10.4
Educational level, n (%)	n = 52
Primary school	21 (40)
Secondary school	10 (19)
University	21 (40)
Marital status, n (%)	n = 54
Married or living together	38 (70)
Living alone; have a partner	1 (2)
Living alone; single or widow/widower	15 (28)
Born in the country of the study, n (%)	n = 54
Yes, born in Sweden	50 (93)
No, born in another European country	3 (6)
No, born in a non-European country	1 (2)
Cancer type, n (%)	n = 55
Prostate	20 (36)
Breast	14 (25)
Lung	7 (13)
Head and neck	6 (11)
Colorectal	4 (7)
Gynecological	3 (5)
Sarcoma	1 (2)
Accumulated radiotherapy dose at T1/Planned total dose, mean ± SD Gy	28.9 ± 18.5 /57.7 ± 16.3
Concomitant chemotherapy during the radiotherapy period, n (%)	n = 55
No	35 (64)
Yes	20 (36)
Activities of daily living,^a^ n (%)	n = 55
Managed all daily activities	29 (53)
Managed more than half of daily activities	15 (27)
Managed approximately half of daily activities	5 (9)
Managed less than half of daily activities	4 (7)
Managed no activities	2 (4)
Number of days practicing physical activity before cancer,^b^ md, 25th to 75th percentile	
Vigorous physical activity	0-0, 0.0-0.0
Moderate physical activity	2, 0-4
Quality of life,^c^ md, 25th-75th percentile	5, 4-6

Number and percent (%) of answering patients are presented.

Abbreviations: n, number; SD, 1 standard deviation; Gy, Gray.

a The past week preceding T1, Time 1.

b Within a regular week the year preceding cancer.

c Graded 0 (worst possible) to 7 (best possible).

Measuring kinesiophobia: Guided by the fear-avoidance model,^19,20^ the T1 questionnaire and the T2 questionnaire covered the Swedish version of the TSK-Symptoms, measuring kinesiophobia in relation to patients’ multiple symptoms during or after cancer therapy. The TSK-Symptoms instrument is our modification of the Swedish version (TSK-SV)^33^ of the TSK,^32^ originally developed for patients experiencing pain. The TSK-SV has shown high reliability (eg, ICC = 0.91; Cronbach’s alpha = 0.81) and been judged to have satisfactory face validity and content validity in a Swedish pain population.^33^ Based on interviews with patients with cancer (n = 11, non-published data), we slightly modified TSK-SV for patients with cancer, demonstrating the great heterogeneity of multiple concurrently occurring symptoms.^2^ The TSK-Symptoms demonstrated good psychometric properties when the measure was tested in 440 patients undergoing cancer therapy (non-published data).^36^ During the modification procedure, the word “pain” was changed to “symptoms” in all items. One typical example of the modification of the TSK-SV to the TSK-Symptoms is: *“No one should have to exercise when he/she is in* ***pain****”* was modified to *“No one should have to exercise when he/she has* ***symptoms****.”* The TSK-Symptoms, like the TSK-SV,^33^ includes 17 items. Each item is rated on a 4-point Likert scale with scoring alternatives ranging from “strongly disagree” to “strongly agree.” A total sum is calculated after the reversal of items 4, 8, 12, and 16. The score ranges between 17 and 68; a higher score indicates higher degree of kinesiophobia.^33^

Measuring various symptoms: The T1 questionnaire and the T2 questionnaire comprised valid and reliable self-assessment of the following symptoms: nausea: “Have you experienced nausea?” (“No, not at all,” “Yes a little,” “Yes moderate,” “Yes, severe”)^22^; pain: “Have you experienced pain in your body?” (“No,” “Mild,” “Moderate,” or “Severe”); anxious and depressed mood: “Have you experienced anxiety?” and “Have you felt depressed or in a depressed mood?” (both 8-graded scales ranging from “0; Never” to “7; All the time”).^37^

Measuring physical activity: Guided by the fear-avoidance model,^19,20^ the T1 and the T2 questionnaires covered valid and reliable self-assessed measuring of physical activity.^38^ The patients provided information on number of days, and number of minutes (using fixed response alternatives) spent practicing moderate and vigorous physical activity during a regular week (defined in the questionnaire as a regular week in the year prior to cancer) and during the week preceding the T1 questionnaire and the T2 questionnaire, respectively.

Qualitative interviews conducted while responding to the study questionnaire: Since kinesiophobia in relation to multiple symptoms in patients undergoing cancer therapy is partly a new research area, an exploratory approach was required, adding qualitative interview data^39^ to the quantitative TSK-Symptoms questionnaire data. This is a common approach in preparatory studies.^34^ A physiotherapist (experienced in cancer care, with previous experiences of conducting interviews) individually, using a semi-structured interview guide, interviewed the first 8 patients when they responded to the study questionnaire T1. The interview guide included one open question: “Could you please describe your own experiences in relation to the topics of the questionnaire?.” The patients responded to the study questionnaire in writing, simultaneous with orally describing their experiences. According to the interview guide, the interviewer made counter questions, for example, “can you tell me a bit more?,” and “how did you experience this?” (referring to the topics of the study questionnaire). At the completion of the interview, the interview guide covered a summarizing question: “We have now been talking about your perception of undergoing treatment, of functioning, quality of life, symptoms, and activities. Is there anything more you want to add regarding your experiences?.” The interviews ranged from 26 to 89 minutes (mean number of minutes 62) and were recorded and then transcribed verbatim. The interviews were conducted within development work in standard care. With just 8 interviewed patients, the intention was not to reach saturation but to reach a deeper understanding of patients’ experiences,^34,39^ to be able to collect data on relevant topics in future full-scale studies and in standard care.

### Statistical Analyses

Descriptive statistics, for example, n and percentages (%) were described regarding the questionnaire data and medical record data. Mean ± one Standard Deviation (SD) described normal frequency distributed interval data (eg, radiotherapy dose). For skewed continuous data (eg, number of days practicing vigorous and moderate physical activity) and ordinal data (eg, anxious mood), we presented median (md) with 25th and 75th percentile. We described feasibility as number (n) of participants who responded to the questions within the study questionnaire, for example each TSK-Symptoms item. No data imputation methods were applied.

We categorized the patients into the groups “Reporting kinesiophobia” and “Not reporting kinesiophobia” based on the cut-off score of >37, as previously defined.^19,33^ We compared the proportions of patients reporting kinesiophobia at T1 and T2 using Chi2 test (nominal variable) and compared the level of kinesiophobia using Wilcoxon’s signed rank test (ordinal variable). Further, we compared the patients reporting nausea (“A little” to “Severe” nausea) to the patients not reporting nausea (“No” nausea) regarding the proportions reporting kinesiophobia (nominal variable) using Chi^2^ test, presented as Relative Risks (RR) with 95% Confidence Intervals (CI). We compared the patients reporting and not reporting pain (“No pain”), anxious mood (rated “0”), and depressed mood (rated “0”), respectively, in the same way. We assigned the patients practicing physical activity (of both vigorous and moderate intensity) at least 1 day during the past week to the group “practiced physical activity” and the patients not practicing physical activity on any day to the group “did not practice physical activity.” Chi^2^ test was used to compare the groups, presented as RR with CI. Further, we compared the patients reporting and not reporting kinesiophobia regarding the non-categorized variables on nausea intensity, anxious and depressed mood, severity of pain (ie, ordinal data), number of days practicing moderate physical activity/vigorous physical activity (ie, continuous but skewed data), using Mann Whitney *U*-test. The significance level was set at 5%, *P* < .05. The SPSS Statistics for Windows (version 24, Armonk, NY: IBM Corp) was used.

### Qualitative Content Analysis of Interview Data

Content analysis^40^ was conducted by the last author (with long experience as a physiotherapist and cancer care researcher, experienced in qualitative analyses). The analysis had a manifest focus, that is, it focused on what experiences the patients described, without making any interpretations of any deeper meaning behind the expressions. The analyzer repeatedly read the transcribed interviews thoroughly, and selected descriptions of kinesiophobia, various symptoms, and physical activity, relevant to the study objective, so called meaning units.^40^ The units should not be too long, avoiding units that could contain more than one phenomenon, nor too short to avoid the risk of fragmented descriptions. Using different codes representing different descriptions,^40^ categories were then initiated. The categories were then discussed with the first author (educated in behavioral medicine, experienced in cancer care research and qualitative analysis), by moving back and forth between the codes and data, in relation to the categories. We presented a descriptive result text for each category highlighted with citations from the patient interviews, according to guidance from methodological literature.^39,40^

## Results

### Description of Participating Patients

Of the 57 patients included, 55 patients provided data, while two did not (Figure 1). Of the 55 participants, most (n = 44; 80%) were 60 to 79 years of age, and approximately half were men (n = 28; 51%). The most common cancer types were prostate (n = 20; 36%) and breast (n = 14; 25%) cancer (Table 1).

### Questionnaire Results on Occurrence of Kinesiophobia

Adherence to the 17 items of TSK-Symptoms is presented in Table 1. Complete responses (100% response rate) were provided on 4 items of the TSK-Symptoms instrument at T2. At T1, the internal attrition ranged between 3 and 9 patients per item. The corresponding figures at T2 were between 1 and 4. Table 2 presents the patients’ responses to the different items of the TSK-Symptoms instrument. As can be seen in Table 3, the proportion of patients reporting kinesiophobia increased (*P* = .009) from 4 of 54 (7%) to 8 of 55 patients (14%) between T1 and T2. At T2, 1 patient no longer reported kinesiophobia, while 5 patients developed kinesiophobia between T1 and T2 (ie, increased level of kinesiophobia to levels above the cut-off was categorized as experiencing kinesiophobia). In the patients reporting kinesiophobia at T1 (n = 4) and T2 (n = 8), the median score of TSK-Symptoms was 42 (25th-75th percentile 40-43) at T1 and 40 (25th-75th percentile 38-43) at T2 (Table 3).

**Table 2.** The Patients’ Responses on the Different Items of Tampa-Scale for Kinesiophobia-Symptoms.

Question	Strongly disagree n (%)	Disagree n (%)	Agree n (%)	Strongly agree n (%)
1. I’m afraid that I might injure myself if I exercise				
Responses at T1 (n = 51)	39 (76)	10 (20)	2 (4)	0 (0)
Responses at T2 (n = 55)	43 (78)	10 (18)	2 (4)	0 (0)
2. If I were to try to be physically active, my symptoms^a^ would increase				
Responses at T1 (n = 51)	38 (74)	7 (14)	4 (8)	2 (4)
Responses at T2 (n = 55)	40 (73)	8 (14)	6 (11)	1 (2)
3. My body is telling me I have something dangerously wrong				
Responses at T1 (n = 50)	31 (62)	9 (18)	6 (12)	4 (8)
Responses at T2 (n = 55)	31 (56)	14 (26)	6 (11)	4 (7)
4. My symptoms^a^ would probably be relieved if I were to exercise^c^				
Responses at T1 (n = 50)	12 (24)	15 (30)	12 (24)	11 (22)
Responses at T2 (n = 54)	18 (33)	13 (24)	11 (20)	12 (22)
5. People aren’t taking my medical condition seriously enough				
Responses at T1 (n = 49)	31 (63)	12 (24)	4 (8)	2 (4)
Responses at T2 (n = 54)	37(68)	11 (20)	6 (11)	0 (0)
6. My symptoms^b^ have put my body at risk for the rest of my life				
Responses at T1 (n = 47)	32 (68)	7 (15)	6 (13)	2 (4)
Responses at T2 (n = 52)	34 (65)	16 (31)	1 (2)	1 (2)
7. Symptoms^a^ always mean I have injured my body				
Responses at T1 (n = 45)	29 (64)	9 (20)	7 (16)	0 (0)
Responses at T2 (n = 54)	31 (57)	17 (32)	2 (4)	4 (7)
8. Just because something aggravates my symptoms^a^ does not mean it is dangerous^c^				
Responses at T1 (n = 47)	6 (13)	8 (17)	16 (34)	17 (36)
Responses at T2 (n = 52)	12 (23)	11 (21)	9 (17)	20 (38)
9. I am afraid that I might injury myself accidentally				
Responses at T1 (n = 50)	35 (79)	12 (24)	3 (6)	0 (0)
Responses at T2 (n = 54)	39 (72)	10 (18)	3 (6)	2 (4)
10. Simply being careful that I do not make any unnecessary movements is the safest thing I can do to prevent my symptoms^a^ from worsening				
Responses at T1 (n = 50)	40 (80)	8 (16)	2 (4)	0 (0)
Responses at T2 (n = 55)	42 (76)	9 (16)	2 (4)	2 (4)
11. I wouldn’t have such severe symptoms^a^ if there weren’t something potentially dangerous going on in my body				
Responses at T1 (n = 46)	24 (52)	11 (24)	8 (17)	3 (6)
Responses at T2 (n = 53)	25 (47)	13 (24)	11 (21)	4 (8)
12. Although my condition is symptomatic,^b^ I would be better off if I were physically active^c^				
Responses at T1 (n = 47)	0 (0)	5 (11)	16 (34)	26 (55)
Responses at T2 (n = 52)	5 (10)	3 (6)	17 (33)	27 (52)
13. The symptoms^a^ let me know when to stop exercising so that I don’t injury myself				
Responses at T1 (n = 50)	19 (38)	8 (16)	16 (32)	7 (14)
Responses at T2 (n = 54)	18 (33)	9 (17)	20 (37)	7 (13)
14. It’s really not safe for a person with a condition like mine to be physically active				
Responses at T1 (n = 48)	35 (73)	6 (12)	6 (12)	1 (2)
Responses at T2 (n = 52)	35 (67)	9 (17)	7 (14)	1 (2)
15. I can’t do all the things normal people do because it’s too easy for me to get injured				
Responses at T1 (n = 49)	38 (78)	5 (10)	5 (10)	1 (2)
Responses at T2 (n = 53)	42 (79)	4 (8)	7 (13)	0 (0)
16. Even though something is causing me a lot of symptoms,^a^ I don’t think it’s actually dangerous^c^				
Responses at T1 (n = 47)	6 (13)	10 (21)	11 (23)	20 (43)
Responses at T2 (n = 51)	8 (16)	12 (24)	15 (29)	16 (31)
17. No one should have to exercise when he/she is having symptoms^a^				
Responses at T1 (n = 48)	19 (40)	9 (19)	11 (23)	9 (19)
Responses at T2 (n = 54)	27 (50)	13 (24)	7 (12)	7 (12)

Abbreviations: TSK-Symptoms, Tampa-Scale for Kinesiophobia-Symptoms; T1, Time 1, conducted after receiving an accumulated dose of mean 28.9 Gy ± Standard Deviation (SD) 18.5 Gy of the total dose 57.7 ± 16.3, and at T2, Time 2, 1 week later (after receipt of additional 5 radiotherapy fractions).

a The word “pain” in the original TSK has in the TSK-Symptoms been changed to “symptoms.”

b The word “accident” in the original TSK has in the TSK-Symptoms been changed to “symptoms.”

c Items 4,8,12 and 16 are reversed when summarizing TSK-S score, as in the original TSK.

**Table 3.** Patients With and Without Kinesiophobia Reporting Various Symptoms and Engagement in Physical Activity at T1 and T2.

Variables	Patients reporting kinesiophobia^a^ at T1 (n = 4)	Patients not reporting kinesiophobia^a^ at T1 (n = 50)	*p*-value	Patients reporting kinesiophobia^a^ at T2 (n = 8)	Patients not reporting kinesiophobia^a^ at T2 (n = 47)	*p*-value
Cancer therapy symptoms, median; 25th to 75th percentile						
Nausea	1.5; 0.25-4.3	0.0; 0.0-0.0	**.012** ^*^	1.5; 0.3-2.8	0.0; 0.0-0.0	**.017** ^*^
Pain	1.0; 0.3-1.0	0.0; 0.0-1.0	.361	1.0; 0.3-1.0	0.0; 0.0-1.0	.317
Anxious mood	0.5; 0.0-3.3	0.0; 0.0-2.0	.833	1.5; 0.0 -2.0	0.0; 0.0-0.0	.050
Depressed mood	1.5; 0.3-2.8	0.5; 0.0-2.0	.534	2.0; 0.3 -2.0	0.0; 0.0-1.0	.071
Physical activity, median; 25th-75th percentile						
Number of days with vigorous physical activity	0; 0-0	3; 2-4	**.026** ^*^	0-0; 0-0	4; 2-4	<.001^*^
Number of days with moderate physical activity	2; 0-4	5; 3-7	**.046** ^*^	2; 0-4	5; 4-7	**.006** ^*^

Symptom experience and physical activity is presented in patients reporting kinesiophobia (score > 37 at TSK-Symptoms) and not reporting kinesiophobia (score < 37) at T1, Time 1, conducted after receiving an accumulated dose of mean 28.9 Gy + Standard Deviation (SD) 18.5 Gy of the total dose 57.7 + 16.3, and at T2, Time 2, 1 week later (after receipt of additional 5 radiotherapy fractions).

a Kinesiophobia was assessed using the instrument TSK-Symptoms, Tampa-Scale for Kinesiophobia-Symptoms.

* Statistically significant difference at 5% significance level (*p*-values are presented in bold text).

### Questionnaire Results on Kinesiophobia in Patients Reporting Nausea, Pain and Anxious or Depressed Mood

**Figure 2.**
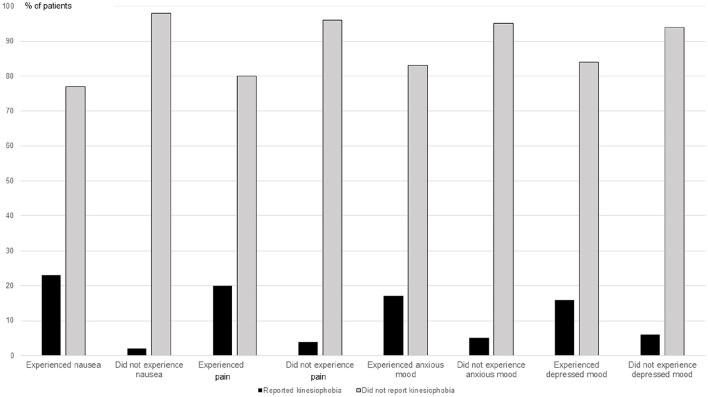
Proportions of patients reporting kinesiophobia (score >37 at the TSK-Symptoms) and not reporting kinesiophobia (score ≤37) of patients with nausea, pain, anxious and depressed mood compared to patients free from these symptoms. Data are presented from n = 54 at T1. Abbreviations: n, number; the TSK-Symptoms, the Tampa-Scale for Kinesiophobia-Symptoms; T1, Time 1, conducted after receiving an accumulated dose of mean 28.9 Gy ± Standard Deviation (SD) 18.5 Gy of the total dose 57.7 ± 16.3.

Of the 13 patients who reported nausea at T1, 3 (23%) reported kinesiophobia. Of the 41 patients who did not report nausea at T1, 1 (2%) reported kinesiophobia (RR 9.5, CI 1.07-83.32, *P* = .039). Of the 16 patients who reported nausea at T2, 6 (38%) reported kinesiophobia. Of the 39 patients who did not report nausea at T2, 2 (5%) reported kinesiophobia (RR 7.3, CI 1.7-32.471, *P* = .005). Some visual inspected differences were indicated regarding also the other studied symptoms (Figure 2). However, patients reporting, or not reporting, pain, anxious mood, or depressed mood, did not statistically significantly differ regarding proportion reporting kinesiophobia.

### Questionnaire Results on Nausea, Pain and Anxious or Depressed Mood in Patients Reporting and Not Reporting Kinesiophobia

At T1 (*P* = .012) and T2 (*P* = .017), the patients reporting kinesiophobia experienced greater intensity of nausea compared to the patients not reporting kinesiophobia (Table 3). The patients reporting and not reporting kinesiophobia did not differ statistically significantly regarding pain, anxious and depressed mood. A non-significant tendency toward higher levels of anxious mood in patients reporting kinesiophobia at T2 was seen (*P* = .050; Table 3).

### Questionnaire Results on Physical Activity in Patients With and Without Kinesiophobia

None of the patients reporting kinesiophobia at T1 (0 of 4) or T2 (0 of 8) practiced vigorous physical activity during the past week. Higher proportions of patients not reporting kinesiophobia practiced vigorous physical activity, 11 of 50 (22%) patients at T1, and 11 of 47 patients (23%) at T2. Three of the 4 (75%) and 6 of the 8 patients (75%) reporting kinesiophobia at T1 and at T2, respectively, practiced moderate physical activity during the past week. Higher proportions of patients not reporting kinesiophobia practiced moderate physical activity: 47 of 50 (94%) patients at T1, and 45 of 47 patients (96%) at T2. The patients reporting kinesiophobia practiced fewer days of both moderate and vigorous physical activity compared to patients not reporting kinesiophobia (Table 3).

### Interviewed Patients’ Experiences

The qualitative content analysis of the interviews resulted in 3 categories describing experiences from the perspective of the patients: “Struggling to stay physically active in an extraordinary situation associated with burdensome symptoms,” “Feeling damaged but at the same time grateful,” and “Needing support due to fear of motion and fear of worsened condition.”

Category “Struggling to stay physically active in an extraordinary situation associated with burdensome symptoms”: The patients described physical activity to be important to them, for providing health effects, and to compensate for the cancer-therapy induced and sickness absence induced inactivity. Being physically active was perceived to be beneficial for both body and mind. However, symptoms such as fatigue, nausea, swollenness, incontinence, and pain, amongst others, were perceived to constitute barriers to engaging in physical activity. The patients struggled hard to overcome these barriers and engage in physical activities. Sometimes they succeed, and sometimes not, depending on how burdensome the symptoms were, and on how much support they felt from healthcare professionals, other patients, and close relatives. The patients described that the cancer therapy per se affected their engagement in physical activity, since the cancer therapy was time consuming and energy consuming, preventing physical activity. The cancer therapy was at the same time a motivation, being the starting point for engaging in physical activity, after being sedentary during the long sickness absence. Inactivity was worsened when having a spouse who took care of all household activities. Close relatives, for example grandchildren, were described to be important facilitators of physical activity.

“It it will be nothing now when I am on sick leave. If I am not even going out for a stroll” (woman, 51 years old, on full time sickness absence, breast cancer)

“Maybe I haven’t gone that fast, but I’ve been out at least” (Respondent 8, man, 64 years old, prostate cancer)

“Even if I swell, it should *not* stop me from exercising (Respondent 5, woman, 72 years old, retired, multiple cancer diagnoses)”

“You just want to rest, you’re so tired” (Respondent 7, man, 45 years old, unemployed, head neck cancer)

Category “Feeling damaged but at the same time grateful”: There were descriptions on experienced harms and negative consequences of the cancer and its therapies. At the same time, the patients expressed gratitude regarding their receipt of beneficial medical cancer therapies. They were grateful for the given rehabilitation or other non-medical cancer care procedures that were perceived as health-promoting and, in a positive way, distracted from the cancer illness and the bothering medical cancer therapies.

“You kind of drag yourself here because you know you’ll feel better afterward” (Respondent 5, woman, 72 years old, retired, multiple cancer diagnoses)

“So, it’s nice to get out and think about something else” (Respondent 1, woman, 51 years old, on full time sickness absence, breast cancer)

Category “Needing support due to fear of motion and fear of worsened condition”: Advice from co-patients regarding engaging in physical activity for managing symptoms were perceived to be helpful, since the co-patients had their own trustworthiness experiences to rely on. There were descriptions on lack of advice and support from cancer care professionals regarding physical activity. However, there were also reflections on that even if they had received advice and carefully listened to them, they would not have adhered to them anyhow, due to their poor condition and a multitude number of symptoms. The patients needed support to overcome fear and uncertainty regarding motion since they had been sedentary for a long time and did not know if and how much they dared to be physically active. Even patients who were currently doing well worried about future side effects and the worries per se could impair their perceived quality of life.

“I’ve been *so* immobile - I don’t know how much I dare to move and how much I dare to strain myself” (Respondent 1, woman, 51 years old, on full time sickness absence, breast cancer)

“Information about physical activity? Not much. . .//. . . However, I don’t think it would have stressed me out. I won’t adhere to them anyhow. Because I couldn’t bear it” (Respondent 7, man, 45 years old, unemployed, head neck cancer)

## Discussion

This preparatory study found the new instrument TSK-Symptoms to be feasible for use in patients with cancer to quantify kinesiophobia, which was present in approximately 1 in 10 patients. Kinesiophobia was more common in patients with nausea, and patients reporting kinesiophobia practiced less physical activity compared to others. Patients undergoing cancer therapy described experiences of struggling to stay physically active in an extraordinary situation associated with burdensome symptoms. They felt damaged but at the same time grateful when considering the impact of the cancer therapies and care and highlighted their need of support due to fear of motion and fear of worsened condition.

The proven feasibility of the new instrument TSK-Symptoms for quantifying kinesiophobia in relation to multiple symptoms was gratifying, since previous studies indicated that assessing and highlighting kinesiophobia in the target population is beset with difficulties.^4,30^ The observation that as many as approximately 10% of patients reported kinesiophobia in relation to their multitude of symptoms surprised us. The sample reflected standard care patients and was accordingly highly heterogenous, consisting not only by symptomatic patients but also of patients not reporting, or reporting discrete symptoms. Despite this, as many as 1 in 10 reported kinesiophobia. Previous studies found rather similar kinesiophobia gradings in patients who all had at least one specific symptom, that is, pain,^5,6,8,31^ fatigue^10-13^ and lymphedema.^15-18^ Given the solid scientific evidence for health effects of physical activity^24,25^ and low patient adherence to physical activity,^26^ the findings of the present study are important in that they reveal that kinesiophobia can be a significant barrier to physical activity. The finding that the fear of motion may be so severe in patients undergoing cancer therapy that it could be considered kinesiophobia is in line with patient experiences^4^ and proposals from researchers and healthcare professionals.^30^ However, in the cited research^4,30^ kinesiophobia was not quantified using any instrument, which seems indispensable for longitudinal evaluations of intervention efforts in cancer care.

A novel finding was the association between presence of nausea and occurrence of kinesiophobia, which was not expected in the light of that previous studies have focused mostly on the relevance of pain and other symptoms for developing kinesiophobia.^5-8,10-13,15-18,31^ Nausea is still ranked among the most incapacitating side effects of cancer therapy.^2^ Besides the considerable symptom burden associated with nausea,^2,21,22^ our study reveals further reasons for identifying and adequately treating patients at risk of experiencing nausea during cancer therapy.^41^ Satisfactory antiemetic treatment may potentially prevent patients from developing nausea-related kinesiophobia and the associated risk of subsequent physical inactivity, which in turn may worsen symptoms. Patients (n = 230) practicing home-based or supervised aerobic physical activity reported dramatically less chemotherapy induced nausea than did patients randomized to standard care.^42^ Further, 66% of 200 patients who practiced physical activity to prevent radiotherapy-induced nausea remained free from nausea during the entire radiotherapy period. Of patients not practicing physical activity, only 9% remained free from nausea.^43^ A daily single-item question has been seen to successfully identifying patients experiencing nausea during emetogenic radiotherapy.^22^ This proactive clinimetric approach^44^ may well lead to adequate antiemetic treatment in more patients and, accordingly, to fewer patients developing kinesiophobia^1^ and avoiding physical activity^19,20^ due to their fear of aggravated nausea and vomiting during physical activity.

In the present study, patients reporting kinesiophobia were less physically active than were patients not reporting kinesiophobia. In line with this, more kinesiophobia in relation to fatigue was previously reported in physical activity program decliners than in patients who adhered.^13^ Our finding was in line with previous findings regarding non-cancer populations^19,20,45^ and in cancer survivors with pain,^5^ fatigue,^13^ or lymphedema.^15,17^ The present finding was expected since it confirms the fear-avoidance model, on avoidance of physical activity when experiencing pain,^19,20^ to be valid even in patients experiencing cancer-related symptoms. During cancer therapy, patients with cancer-related symptoms may perceive physical activity, which they tolerated prior to cancer therapy, as an excessive burden. This may cause insecurity, fear, and catastrophic thinking about the possibility that physical activity might aggravate the symptoms. Paradoxically, if their response is to further avoid physical activities, instead of gradually exposing themselves to physical activity, the symptoms may persist. In contrast, exposure to physical activity may instead provide opportunities to feel the positive effects of physical activity.^19,20^ Sufficient physical activity pre breast cancer surgery did not prevent kinesiophobia post surgery, indicating the need for supporting patients when undergoing cancer therapy irrespectively of their ordinary activity level.^9^ The insufficient level of physical activity in patients with kinesiophobia observed in our present study highlights the need for support, as support from cancer care professionals could allow patients to overcome this barrier to physical activity.^4^ Evidence-based interventions reducing kinesiophobia during cancer therapy are still lacking. However, 2 systematic reviews of practicing Pilates or of receiving pain neuroscience education to reduce kinesiophobia documented promising results in non-cancer patients with low back pain,^46^ and neck pain.^47^ It is not known whether these interventions can be effectively transferred to patients with a multitude of simultaneously occurring cancer-related symptoms. Two randomized studies using Kinect-based virtual reality therapy,^5^ or mirror therapy,^48^ respectively, in women undergoing breast cancer therapy showed promising results on kinesiophobia in relation to pain. Likewise, a multimodal exercise rehabilitation program decreased kinesiophobia in relation to fatigue.^14^ However, replicating studies are needed since just 20, 34, and 24 patients received the interventions.^5,14,48^ We believe that intervening therapists can use a kinesiophobic patient’s responses to the TSK-Symptoms instrument to identify areas associated with need for patient education and gradual exposure to motion. This might hopefully increase the patient’s trust in that physical activity would not induce worsening of his/her burdensome symptoms.

At the time for the T2 TSK-Symptoms questionnaire, there was a non-statistically significant tendency for patients reporting kinesiophobia to report more anxious mood than patients not reporting kinesiophobia. This potential association between anxiety and kinesiophobia makes sense, as anxiety is the primary affective component of phobias^49^ and a relation between kinesiophobia and anxious mood has been established in breast cancer survivors.^15^ Previous studies have also demonstrated the influence of anxiety on kinesiophobia among patients with chronic musculoskeletal pain.^45^ Although long-term persistent pain was previously reported to be associated with kinesiophobia,^6,15^ we observed no statistically significant difference in pain between patients reporting versus those not reporting kinesiophobia in the current study. Based on knowledge generated by studies grading distress caused by symptoms,^2^ our participants may have experienced a higher symptom burden of nausea than of pain, possibly explaining the lack of an identified association between pain and kinesiophobia in our study. Another reason may be lack of statistical power due to the rather small sample size.

Interestingly, the interviewed patients undergoing cancer therapy described experiences of being struggling to stay physically active in the extraordinary situation of undergoing cancer therapy, associated with multiple burdensome symptoms. Engagement in physical activity did not come naturally when being on sickness absence due to the time-consuming and often burdensome cancer therapy. The patients felt damaged but at the same time grateful when considering the impact of the cancer therapies and care, and described they needed support due to fear of motion and fear of worsened condition. These experiences had similarities with how persons who previously had undergone chemotherapy described their situation.^50,51^ They perceived physical activity to be beneficial for health and frequently tried to practice physical activity to the best that they possibly could manage while affected by painful unpleasant symptoms^50^ or nausea.^51^ The described damage, fear, and insecurity on if and how much to be physically active was in line with the fear-avoidance model; fear of motion may induce a sedentary behavior. The patients did not use the body for what it is made for, which in turn leads to worsened functional and activity impairment.^20^ This highlights the need for support early during the phase of undergoing cancer therapy, to avoid persistent insufficient physical activity and kinesiophobia in the long-term, which has been reported in breast cancer survivors^52^ and in various survivors who previously have had an implantable venous access port.53^8^

Concerning the methodology used in the present preparatory study, we in line with the literature on preparatory studies^34^ studied several aspects of the feasibility of the study logistics and study questions, while results regarding psychometric properties of the TSK-Symptoms instrument will be reported elsewhere (n = 440 patients, non-published data). We found the process to be feasible and the personnel and patient time expenditures (eg, the retention and refusal rates) to be reasonable. Further, we improved our understanding of the study questionnaire, specifically the TSK-Symptoms instrument, and provided a deeper understanding on the patients’ experiences by interviewing some of them when responding to the study questionnaire. The response rate of 98% was highly satisfactory, and the internal attrition was reasonable. Regarding study management, we found that the radiotherapy nurses had enough time to screen patients for the study criteria as well as to provide the study information and study questionnaire to the patients. Finally, the present preparatory study applied a theory guided data collection^19,20^ adhering to clinimetric criteria for patient-reported outcome measures^44^ to be able to evaluate several scientific aspects^34^ preceding the planned full-scale study. This involved producing preliminary results on the occurrence of kinesiophobia as well as on potential differences between patients reporting and not reporting kinesiophobia. We chose a cut-off score of >37 on the TSK-Symptoms scale to denote kinesiophobia, following the original definition based on TSK values in patients with chronic musculoskeletal pain.^19^ In the present study, this cut-off score was used to make our results comparable. However, in a clinical application, the issue of cut-off scores needs to be further analyzed for patients with cancer, as TSK scores may differ across populations.^53^ To avoid potential therapist-induced bias, the well-informed^54^ patients were asked to complete the questionnaire in private and not at the radiotherapy department. We, guided by the fear-avoidance model^19,20^ collected data on kinesiophobia, daily^21^ and physical activity^38^ as well as symptoms^22,36,37^ from the patient’s perspective,^44^ because gaps often exist between patients’ and cancer care professionals’ estimations of the burden of the patient’s health problems.^55^ The study group was heterogeneous, including male and female patients being treated for a variety of cancer types at various stages, with and without concomitant chemotherapy or other cancer therapies, reflecting patients undergoing standard cancer care.

Valuing the study limitations, this preparatory study naturally collected questionnaire data and interview data from a small number of patients. Collecting data from a smaller number of respondents over a limited period before a full-scale study is the essence of a preparatory study. This contributes to test of the study procedures’ feasibility and to provide preliminary results.^34^ No sample size calculation was conducted, and the small sample size plausibly induced a type II error, explaining that the visually inspected differences in presence of various symptoms did not reach statistically significance, except for nausea. For example, the absolute median scores regarding anxious and depressed mood were worse in the kinesiophobic patients at T2 but the worse gradings did not reach statistical significance (*P* = .05, *P* = .71) at a 5% significance level. Due to the small number of participants, we did not analyze whether there were any clinical, morbidity, age- or sex-related differences in symptoms, physical activity or kinesiophobia. The study lacked statistical power to conduct a multivariable regression model analyzing if the variables nausea and insufficient engagement in physical activity were still valid to contribute to the variation in the TSK-Symptoms score after controlling for these descriptive variables. A full-scale study is needed to study kinesiophobia in the target population, allowing for subgroup analyses and multivariable analyses. Regarding the qualitive interviews, the goal was not to reach criteria for saturation with just 8 interviewed respondents. The intention by interviewing some questionnaire-responding patients was rather to understand the patients’ experiences and perceptions,^34,39^ to secure that the study questionnaire did not lack important topics.^34^ This goal was obtained; for example we learned that future study questionnaires may include items on work activity, not only daily activity. More in-depth qualitative interviews are suggested for future research to gain deeper understanding of patients’ experiences. Further studies should include more details regarding the multiple symptoms experienced during cancer therapy, for example by studying a variety of dimensions of single symptom, including symptom burden, and by analyzing symptom clusters. Another limitation is the small number of time points for data collection; a greater number of repeated measures would have been preferable. However, to limit the burden of the data collection on the patients in this preparatory study, we chose to apply just 2 time points. Further, bias due to attrition tends to increase with higher number of time points for data collection. We did not follow up patients after completed radiotherapy. Since kinesiophobia may be persistent,^8,52^ a long-term follow-up would be preferable in future full-scale studies. Physical activity is commonly measured using self-reported data.^38^ In our study, this naturally entails the risk of recall bias and over- and underestimation of physical activity, which would have been eliminated using accelerometry measures. However, we felt that accelerometry measures would have put too great burden on the patient. Further, the threat to the internal validity of our study due to recall bias and social desirability seems to be limited since patients with kinesiophobia would probably not differ from patients without kinesiophobia in recall bias and social desirability when self-reporting physical activity. Thus, it is reasonable to assume that these factors do not explain the observed physical activity differences between patients with and without kinesiophobia. The small number of participants and the rather high mean age of 67.5 years lowers the generalizability, especially to young patients or to patients with other cancer types, undergoing other cancer therapies.

We agree with the recently updated physical activity and cancer control framework proposing that future research should pay attention to various patient populations at risk for insufficient engagement in physical activity.^56^ For example, patients with kinesiophobia need more support from cancer care professionals than others to be able to engage in sufficiently levels of physical activity. According to the findings of the current preparatory study, we imply that the new TSK-Symptoms instrument is feasible for quantifying kinesiophobia in relation to the multitude of symptoms among patients undergoing cancer therapy in standard care and in future studies. Studies with greater sample sizes, focusing on, for example, the psychometric properties of the TSK-Symptoms in several languages, the prevalence of, risk factors for, and interventions for kinesiophobia during and after cancer therapy are welcomed. Despite of the limitations naturally coming with a preparatory study, the study is still innovative, contributing to a feasible instrument for quantifying kinesiophobia in relation to multiple symptoms in patients with cancer.

Since kinesiophobia was present in as many as approximately 1 in 10 patients undergoing cancer therapy and kinesiophobia was associated with less engagement in health-improving and life-prolonging physical activity, this implies that cancer care professionals may further identify and quantify kinesiophobia using for example, the TSK-Symptoms instrument and give kinesiophobic patients support.
